# Preclinical assessment of a novel small molecule inhibitor of indoleamine 2,3-dioxygenase 1 (IDO1)

**DOI:** 10.1186/2051-1426-2-S3-P195

**Published:** 2014-11-06

**Authors:** Gregory Driessens, Stefano Crosignani, Michel Detheux, Benoît Van den Eynde, Sandra Cauwenberghs

**Affiliations:** 1 iTeos Therapeutics, Gosselies, Belgium; 2 Ludwig Institute for Cancer Research (LICR, Brussels Branch) and de Duve Institute, Université catholique de Louvain (UCL), Brussels, Belgium

## Introduction

iTeos leverages the science of the LICR to target the metabolism of the tumor microenvironment and develops small-molecule inhibitors. Tryptophan catabolism can suppress the anti-tumor immune response through expression of the rate-limiting enzyme IDO1. Local tryptophan reduction and metabolite production by the kynurenine pathway are associated with anergy/apoptosis of tumor-infiltrating lymphocytes. Two IDO1 inhibitors (Incyte INCB24360/NewLink NLG919) are currently tested in clinical trials for treatment of relapsed/refractory solid tumors but exhibit therapeutic limitations.

## Methods/results

A primary HTS (176,000 compounds) led to the discovery of several confirmed hits. Medicinal chemistry optimization resulted in an original lead with nM potency in a relevant human blood assay, comparable to Incyte IDO1_i _(IC_50 _890 ± 220 nM [iTeos IDO1_i_] versus 970 ± 100 nM [Incyte IDO1_i_] [N = 3 donors; Kyn ELISA]). For rodents & monkey, iTeos compound showed low clearance (9.9&1.9 mL/min/kg respectively), moderate-to-low protein binding (48&28%Fu respectively), a short half-life in rodents but long in monkey (1.1&12 h respectively), good oral availability (>75%), and no inhibition on five CYP450 isoforms). Good predictive values for human PK were obtained through allometric scaling. Quantification of the tryptophan metabolite kynurenine showed a significant decrease in mouse plasma after oral administration of the IDO1_i _in a dose response setting (45.3 ± 2.4 [iTeos IDO1_i_] and 48.1% ± 3.0 [Incyte IDO1_i_]; 2 h per os at 100 mg/kg compared to vehicle; N = 5 independent experiments). In a time-course experiment, inhibition of kynurenine production lasted 8 h (iTeos IDO1_i_) compared to 16 h (Incyte IDO1_i_), consistent with their respective rodent half-life. A comparable PD effect was observed in mouse tumor lysates. Monkey PD is ongoing. Preliminary data obtained with the Pan02 mouse tumor model showed significant survival benefit of the iTeos IDO1_i _in stand-alone and in combination with anti-CTLA4 treatment compared to vehicle or anti-CTLA4 in stand-alone respectively. 15 different human cancer types (60 samples each) were analyzed for IDO1 protein expression and allowed identification of several tumors with high expression.

## Conclusions

iTeos' drug discovery efforts have delivered a selective IDO1 small-molecule inhibitor with novel scaffold suitable for *in vitro*/*in vivo *validation and with good predictive human PK (low clearance(<10% Q_H_), long half-life(>20 h), high oral bioavailability).

